# A case of yogurt central line‐associated bloodstream infection in a child with intestinal failure

**DOI:** 10.1002/ncp.70025

**Published:** 2025-08-30

**Authors:** Nasiha Rahim, Emily Keller, Ellen Wagner, Andrea Berkemeyer, Timothy Johnson, Daniel Johnson, Timothy Sentongo

**Affiliations:** ^1^ Section of Gastroenterology, Hepatology & Nutrition, Comer Children's Hospital University of Chicago Chicago Illinois USA; ^2^ Division of Infectious Diseases Advocate Children's Hospital, Advocate Health Care Oaklawn Illinois USA; ^3^ Division of Gastroenterology, Hepatology & Nutrition, Lurie Children's Hospital Northwestern University Chicago Illinois USA; ^4^ Dept of Veterinary and Biomedical Sciences University of Minnesota St Paul Minneapolis USA; ^5^ Section of Infectious Diseases, Comer Children's Hospital University of Chicago Chicago IIllinois USA

**Keywords:** central line‐associated bloodstream infection, intestinal failure, *Lactobacillus*, short bowel syndrome, yogurt

## Abstract

Central line‐associated bloodstream infections (CLABSI) are a significant cause of morbidity in children with intestinal failure (IF). Probiotic therapy is discouraged in patients with IF because of the increased risk of bacteremia with the probiotic organism. We report an unusual, previously undescribed, clinical presentation of a *Lactobacillus*‐species CLABSI linked to yogurt consumption in a toddler with IF secondary to Megacystis–microcolon–intestinal hypoperistalsis syndrome. *Lactobacillus* is abundant in fermented foods like yogurt and among the commensal constituents of the oral cavity microbiome and mucosal surfaces in the gastrointestinal tract. It is rarely implicated as the causative organism in CLABSI. At the time of presentation with fever, blood cultures were collected from the patient's central venous catheter (CVC), and peripherally. Both grew *Lactobacillus* species. Comparative genetic analysis predicted ~99% similarity between the CLABSI isolate and *Lactobacillus* isolates from the Greek yogurt the patient regularly consumed. Our patient's *Lactobacillus* CLABSI was linked to the consumption of Greek yogurt. We speculate that daily consumption of yogurt with live cultures posed a high cumulative exposure to *Lactobacillus* species either through external contamination of our patient's CVC or translocation of the ingested organisms from his gastrointestinal tract into the bloodstream. This is the first case report that links CLABSI in children with IF to yogurt consumption.

## CASE REPORT

A 29‐month‐old toddler with intestinal failure (IF) due to Megacystis–microcolon–intestinal hypoperistalsis syndrome (MMIHS)/myopathic intestinal pseudoobstruction syndrome, ileostomy, gastrostomy tube, and central venous catheter (CVC) for supplemental nutrition with parenteral nutrition (PN) providing 50% of his daily fluid, protein and calorie requirements presented to Comer Children's Hospital emergency department with a 1‐day history of fever to 101°F, raising concerns for central line–associated bloodstream infection (CLABSI). He underwent a sepsis work‐up that included blood cultures drawn from his CVC and peripheral vein, then immediately started on intravenous (IV) antibiotic therapy with vancomycin and ceftriaxone.

His oral diet consisted of regular table food as tolerated and daily consumption of 16–24 oz/day of Greek yogurt. His family and medical providers welcomed and encouraged the increased consumption of Greek yogurt because of its high protein content.^1^ His daily medications included trimethoprim‐sulfamethoxazole for urinary tract infection prophylaxis, loperamide as needed to control dumping symptoms, and ferrous sulfate for microcytic anemia (Hgb 9.8 g/dl; MCV 69 fL), and no current or prior use of probiotics. A private home duty nurse (RN) performed weekly line dressing, and the parents daily connected and disconnected his PN infusions with strict adherence to aseptic protocol.

The patient's fever defervesced within 24 h of initiating IV antibiotics. The peripheral and central blood cultures grew a Gram‐positive rod within 25 h in both the aerobic and anaerobic bottles, which was subsequently identified as *Lactobacillus* species. Therapy with Vancomycin was discontinued,^2^ and serial follow‐up blood cultures throughout the rest of his 1‐week hospital stay remained negative. Furthermore, because of the identification of *Lactobacillus*, which is typically a low‐virulent organism,^3^ and the dietary history that he regularly consumed large servings (16–24 oz/day) of Greek yogurt, there was concern about a potential microbial link between the CLABSI and his dietary exposure. Therefore, the parents were instructed to refrain from serving him the Greek yogurt or probiotics while further investigation was conducted to determine whether there was a microbial link between the CLABSI isolate and the yogurt that he regularly consumed.

Comparative isolate cultures and genomics were performed to determine whether the CLABSI‐causing *Lactobacillus* isolates matched the Greek yogurt's live cultures. The exact yogurt cup from which the patient ate was no longer available; however, the family supplied representative cups of the same brand of Greek yogurt purchased on three different dates and a different store brand of yogurt (brand X) for comparison. The Greek yogurt grew *Lactobacillus* species. The culture results are shown in Table 1. Whole genome sequencing was performed on CLABSI isolate and compared with the Greek yogurt isolates. The CLABSI and Greek yogurt *Lactobacillus* isolates matched except for a single nucleotide variant between isolates, suggesting that the organism isolated from the patient's blood was the same as the one contained in the Greek yogurt. Based on the comparative genomics that showed a striking resemblance between CLABSI and Greek yogurt isolates, the instructions to avoid yogurt live cultures or therapy with probiotics were reinforced. The family complied with the recommendation, and the patient has not had a recurrence of *Lactobacillus* CLABSI in 5 years.

**Table 1:** Bacteria isolates cultured from blood and yogurt samples.

Isolate number	Description	Organisms cultured
Isolate #1	Peripheral culture	*Lactobacillus* species
	CVC culture	*Lactobacillus* species
Isolate #2	Greek yogurt, Date #1	*Streptococcus salivarius* ssp. *ThermophilusLactobacillus* species
	Greek yogurt, Date #2	*Streptococcus salivarius* ssp. *ThermophilusLactobacillus* species
	Greek yogurt, Date #3	*Lactobacillus* species
Comparison yogurt	Yogurt Brand X, Date #1	*Streptococcus salivarius* ssp. *Thermophilus*
	Yogurt Brand X, Date #2	*Streptococcus salivarius* ssp. *Thermophilus*
	Yogurt Brand X, Date #3	*Streptococcus salivarius* ssp. *Thermophilus*

Abbreviation: CVC, central venous catheter.

## METHODS

Our institutional review board waived the requirement of consent for anonymized single‐patient case reports.

### Patient blood culture protocol at presentation

Two sets of blood cultures were collected from the patient in BACTEC Peds Plus Aerobic/F and Standard Anaerobic/F vials. The vials were incubated on the BACTEC FX instrument. All four vials were flagged positive within 25 h. A Gram‐stain of the vials demonstrated elongated Gram‐positive bacilli. The organism grew on both trypticase soy agar with 5% sheep blood (BAP) and chocolate agar plates. Colonies were gray in color and produced alpha‐hemolysis on the BAP. No growth was observed on the anaerobic Brucella plates. Identification was performed using the Vitek MS MALDI‐TOF (bioMérieux) using an in vitro diagnostics (IVD) database. The software was unable to distinguish between the following *Lactobacillus* species: *Lactobacillus rhamnosus*, *Lactobacillus paracasei*, and *Lactobacillus casei*. The culture result was reported as “*Lactobacillus* species.”

### Yogurt cultures

The yogurt was plated onto trypticase soy agar with 5% sheep blood (BAP), chocolate, and Brucella agar plates. The BAP and chocolate agar plates were incubated in 5% CO_2_ at 35°C. The Brucella agar plate was incubated anaerobically for 48 h prior to examination. The organisms shown in Table 1 were isolated aerobically and identified using the bioMérieux Vitek MS MALDI‐TOF instrument and the IVD database. No anaerobes were isolated.

### Sequencing

Sequencing was performed at the University of Minnesota Genomics Center. Isolates were grown overnight from a single colony in de Man–Rogosa–Sharpe broth with shaking. DNA was extracted from each isolate using the Qiagen DNEasy kit according to the manufacturer's instructions. Nextera libraries were created using genomic DNA. Sequencing was performed using a single 250‐base pair (bp) dual index run on an Illumina MiSeq to generate approximately 50‐to‐100–fold coverage per genome.

Genome assembly of MiSeq reads was performed using CLC Genomics Workbench, version 7.5 (CLC Bio). Assembly parameters were as follows: automatic word size, automatic bubble size, minimum contig size of 500 bp, length fraction of 0.5, and similarity fraction of 0.8. Genome annotation was performed using the National Center for Biotechnology Information (NCBI) Prokaryotic Genomes Automatic Annotation Pipeline (PGAAP: http://www.ncbi.nlm.nih.gov/genome/annotation_prok/). Variant detection was performed by mapping raw reads from each isolate to the draft assembly of the other isolate. Mapping was performed in CLC Genomics Workbench with the following parameters: length fraction of 0.5, similarity fraction of 0.9, ignoring mapping of nonspecific matches. After mapping, basic variant detection was performed with the following parameters: minimum coverage of 20, maximum coverage of 1000, minimum frequency of 90%, and ignoring broken pairs.

For analysis of gene content, the assembled genomes were uploaded to the RAST Server for automated annotation and comparisons between strains.^4^ The genomes were compared with each other using two‐way protein BLAST with a cutoff of 90% amino acid similarity across 90% of the predicted protein. Genome alignments were also performed in MAUVE.^5^

## RESULTS

### Blood cultures

The central and peripheral blood cultures (Isolate #1) both grew within 25 h. A Gram‐stain of the vials demonstrated elongated Gram‐positive bacilli. The culture result was reported as *Lactobacillus* species. The culture identification software was unable to distinguish between *Lactobacillus rhamnosus*, *Lactobacillus paracasei*, and *Lactobacillus casei*.

### Greek yogurt

Two of the three batches of Greek yogurt (Isolate #2) purchased different dates grew *Streptococcus salivarius* ssp *Thermophilus* and *Lactobacillus* species. One batch of the Greek yogurt only grew *Lactobacillus* species (see Table 1).

### Comparison yogurt (brand X)

The three batches of comparison store brand yogurt (Isolate #3) purchased on different dates all grew only *Streptococcus salivarius* ssp *Thermophilus* (see Table 1).

### Comparative genomics

When reads from Isolate #1 (the clinical isolate) were mapped against the assembly of Isolate #2 (the Greek yogurt isolate), one single nucleotide variant was identified as different between the two genomes. This nonsynonymous change resulted in an amino acid change from proline to threonine in a predicted hypothetical protein. The exact change was identified when mapping reads from Isolate #2 against the assembly of Isolate #1. Four additional single nucleotide pairs were initially identified in this mapping, but all occurred at the ends of contigs and were deemed to be due to sequencing error after further analysis.

The assembled 47 contigs of Isolate #1 (clinical isolate) totaled 2,943,624 bp in length, and the assembled 56 contigs of Isolate #2 (Greek yogurt) totaled 2,945,325 bp in length. MAUVE was used to compare the genome assemblies of isolates #1 and #2, and no large‐scale polymorphisms or gene content differences were identified. For comparison purposes, isolates were also mapped to *Lactobacillus rhamnosus* strain GG (GenBank AP011548.1). However, the isolates differed from strain GG by 50,474 and 51,307 variants, respectively, and are thus not genetically related to strain GG. Therefore, we inferred that the *Lactobacillus* strain causing the CLABSI, and in the Greek yogurt was not *Lactobacillus rhamnosus* GG.

## DISCUSSION

Therapy with probiotics in patients with short bowel syndrome and indwelling CVCs has been associated with an increased risk of bacteremia with the same probiotic organism.^6^, ^7^ Therefore, probiotic therapy is discouraged in vulnerable patients with indwelling CVCs.^8^, ^9^ However, the consumption of yogurt by children with IF and indwelling CVC has not been reported to be a potential risk factor for developing CLABSI. We present a compelling clinical case linking *Lactobacillus* CLABSI in a child with IF to the consumption of Greek yogurt. This is the first case report that links the consumption of yogurt with live cultures to CLABSI in a patient with IF and an indwelling CVC. Therefore, we raise a new caution about an increased risk of CLABSI in children with IF and CVC who consume yogurt with live cultures.

We used whole genome sequencing to identify and compare the patient's CLABSI isolate with a representative sample of the Greek yogurt product that he regularly consumed. The *Lactobacillus* isolates were genetically identical except for a single nucleotide variant. *Lactobacillus* species are known to be commensal organisms found in the oral cavity, gastrointestinal tract, and female genitourinary tract. Previous studies have shown that these organisms can be pathogenic, especially in immunocompromised hosts.^10^ The most frequently isolated species are *L. rhamnosus* and *L. casei*.^10^ and the most common clinical infections are bacteremia and endocarditis. The bacteria species identification software available to us could not differentiate *Lactobacillus* species; however, genomic mapping strongly indicated that the CLABSI and Greek yogurt isolates were identical. Furthermore, genome mapping determined that the CLABSI and Greek yogurt isolates were not *Lactobacillus* GG. The standard live cultures routinely used in the manufacturing of yogurt are *Streptococcus thermophilus* and *Lactobacillus bulgaricus*.^1^ Therefore, we suspect that the isolate may have been *Lactobacillus bulgaricus*.

Probiotics typically have a live culture count ≥10^10^ colony‐forming units/g (CFUs/g) compared with fresh yogurt, with live counts ranging from ×10^7^ to 10^9^ CFUs/g.^11^, ^12^ Therefore, a person would need to consume approximately 1000 g (33 oz)/day of yogurt with live cultures to achieve the same *Lactobacillus* exposure as a unit dose of probiotics. Our patient regularly consumed 16–24 oz (480–720 g/day)/day of Greek yogurt for several weeks, which was equivalent to daily exposure to *Lactobacillus* counts of 4.8–7.2 × 10^9^ CFU/day. Increased consumption of live culture yogurts (1–2 L/week) was, in hindsight, identified as the source of recurrent *Lactobacillus bacteremia* and endocarditis in an elderly 90‐year‐old patient.^13^ Therefore, we speculate that daily consumption of Greek yogurt led to cumulatively increased exposure to live cultures of *Lactobacillus* >4.8 × 10^9^ CFU/g/day that led to intestinal colonization with the *Lactobacillus* and, subsequently, bacterial translocation^14^ into his bloodstream and CVC, resulting in the CLABSI. An alternative hypothesis is that our patient's CVC was externally contaminated^8^ with *Lactobacillus* microbes during the daily multiple exposures to yogurt with live cultures.

The only viable culture in the comparison yogurt (Brand X), purchased on different dates, was *Streptococcus salivarius* ssp *Thermophilus*. One (Date #3) of the three batches of the Greek yogurt cultures – all purchased on different dates, only grew *Lactobacillus* species compared with its identical brand that grew both *Lactobacillus* species and *Streptococcus salivarius* ssp. *Thermophilus*. We attributed this difference to a decline in live cultures that may have occurred because of extended shelf‐life or warmed temperature during handling.^15^ Case reports and reviews that associate *Lactobacillus* species with probiotics should be reviewed cautiously, as data suggest that morphological, physiological, and biochemical criteria are insufficient to distinguish isolates.^16^, ^17^ Standard molecular methods, such as pulse‐field gel electrophoresis, antibiotic susceptibility testing, and strain‐specific PCR amplification, may also be inaccurate. Recent recommendations suggest that whole genome sequencing may be necessary to correctly identify identical isolates. On the other hand, phenotypic properties, such as virulence, have been shown to be different in isolates, indistinguishable by molecular methods.^18^

Probiotic therapy is generally avoided in vulnerable patients with CVCs because of the increased risk for CLABSI with the probiotic organism.^8^ CLABSIs are a major cause of morbidity in children with IF. We have demonstrated that our patient with IF acquired a CLABSI from *Lactobacillus* organisms identical to the isolates present in the Greek yogurt he regularly consumed. The North American Society of Pediatric Gastroenterology, Hepatology and Nutrition (NASPGHAN) Intestinal Rehabilitation special interest group published a position paper with recommendations for the management of central venous access and CLABSI in children with IF.^19^ We advise caution regarding the consumption of yogurt with live cultures, as it may be a potential risk factor for CLABSI in children with a CVC for IF. This seemingly rare entity should be considered when discussing dietary restrictions for children with IF. We further propose that dietary options for yogurt in patients with IF should be limited to varieties without live cultures, that is, heat‐inactivated fermented yogurt or the directly acidified variants of yogurt.^1^, ^20^

## AUTHOR CONTRIBUTIONS

Emily Keller and Timothy Sentongo were responsible for the first draft of this manuscript; Nasiha Rahim, Ellen Wagner, and Andrea Berkemeyer were responsible for presentation of the work and revisions of the manuscript; Timothy Johnson performed and interpreted the genomic analyses; Daniel Johnson provided a critical review of the scientific concepts; and Timothy Sentongo fulfilled all roles except for the genomic analysis, provided mentorship, and carried out the final revisions of the manuscript.

## CONFLICT OF INTEREST STATEMENT

None declared.
